# The Dissemination Research Logic Model for advancing dissemination science and practice

**DOI:** 10.1186/s13012-026-01530-2

**Published:** 2026-07-30

**Authors:** Katherine G. Merrill, Kristi E. Gamarel, Sylvie Naar, Lisa B. Hightow-Weidman, Sybil Hosek

**Affiliations:** 1 Center for Dissemination and Implementation Science, Department of Medicine, University of Illinois Chicago, Chicago, IL USA; 2 Department of Health Behavior and Health Equity, University of Michigan School of Public Health, Ann Arbor, MI USA; 3 Population Studies Center, Institute for Social Research, University of Michigan, Ann Arbor, MI USA; 4 Department of Behavioral Science and Social Medicine, College of Medicine, Florida State University, Tallahassee, FL USA; 5 Institute on Digital Health and Innovation, College of Nursing, Florida State University, Tallahassee, FL USA

**Keywords:** Dissemination science, Dissemination research logic model, Implementation research logic model, Adolescent medicine trials network, HIV prevention, Adolescents

## Abstract

**Background:**

Relative to implementation science, dissemination science is at an earlier stage of development and lacks a tool for specifying and visually depicting the components of the dissemination process. Dissemination science studies the strategic spread of information whereas implementation science focuses on the integration of interventions within specific settings. As a counterpart to the Implementation Research Logic Model, we propose the Dissemination Research Logic Model (DRLM) to fill this gap. Our work is informed by the Adolescent Medicine Trials Network for HIV Interventions (ATN), which advances biomedical and behavioral approaches to strengthen HIV prevention and care for adolescents and young adults.

**Methods:**

We define the DRLM’s constructs and sequence and present key principles and best practices for its use. We then apply the model to two ATN studies: (1) Project PrEPare (ATN 113), the first U.S. adolescent pre-exposure prophylaxis demonstration project for adolescent men who have sex with men, and (2) Tailored Motivational Interviewing (TMI; ATN 128 and 146), an adjunct clinic-based motivational interviewing intervention addressing HIV among youth.

**Results:**

We use the DRLM to specify each project’s components, including: what is being disseminated; the intended audience(s); and dissemination determinants, strategies, channels, mechanisms, and outcomes. Project PrEPare illustrates a broad dissemination campaign in which evidence supporting the safety, acceptability, feasibility, and effectiveness of PrEP for adolescents was disseminated to policymakers, healthcare providers, and youth, with dissemination outcomes including perceived fit, leadership endorsement, and decision to implement. TMI provides an ongoing example of disseminating an adjunct intervention to clinic leadership and staff, with the decision to implement TMI representing the primary desired dissemination outcome and a transition point to implementation.

**Conclusions:**

The DRLM offers a flexible tool for visually depicting how dissemination processes unfold. The DRLM can strengthen the design, conduct, and reporting of dissemination studies while complementing broader approaches, such as knowledge mobilization. Future research should test and refine the DRLM across diverse projects and settings to increase conceptual clarity and comparability across studies and to accelerate dissemination science.

**Supplementary Information:**

The online version contains supplementary material available at https://doi.org/10.1186/s13012-026-01530-2.

Contributions to the literature

- Introduces the Dissemination Research Logic Model (DRLM) as a methodological tool to specify and visually link key components of the dissemination process.
- Demonstrates application of the DRLM using two studies from the Adolescent Medicine Trials Network for HIV Interventions (ATN).
- Advances methodological precision by clarifying relationships among dissemination determinants, strategies, channels, mechanisms, and outcomes.
- Identifies opportunities for further advancement of the field.

## Introduction

While implementation science has seen rapid growth over the past two decades, dissemination science has not kept pace [1–3]. Purtle et al. suggest that the field of dissemination and implementation (D&I) science is more accurately denoted as “d&I” with a lowercase ‘d’ [2]. Strengthening dissemination science as a distinct field is necessary to ensure that research evidence reaches the people and systems positioned to act on it, an essential step in closing the gap between research and practice.

Dissemination science is also related to a broader, international field focused on how research evidence reaches and is used in policy and practice [4]. This work is well established in the United Kingdom and Canada, where it is often described as knowledge mobilization [5]. The field of knowledge mobilization has largely focused on the relationships, organizational capacities, and systems that shape whether research is used, complementing the closer attention dissemination science gives to how specific findings reach particular audiences [6, 7].

Dissemination and implementation are related but distinct, and both have numerous definitions in the literature [1, 8]. We draw on Rabin et al.’s definitions of dissemination as “an active approach of spreading evidence-based interventions or knowledge to the target audience via determined channels using planned strategies *”* [9] and of implementation as *“*the process of putting to use or integrating evidence-based interventions within a setting *”* [10]. The dissemination definition distinguishes dissemination from diffusion [11–14], emphasizing deliberate, planned efforts rather than natural, passive spread of information or ideas. Whereas dissemination practice focuses on using dissemination strategies to share evidence with intended audiences [15], dissemination science refers to the scientific study of the dissemination process.

Smith et al.’s Implementation Research Logic Model (IRLM) has notably advanced the field of implementation science by improving the specification, transparency, and synthesis of relationships among implementation determinants, strategies, mechanisms, and outcomes [16, 17]. Both dissemination and implementation can be understood through a program theory lens, which seeks to specify how and why activities produce desired outcomes [18]. Similar to the IRLM, the dissemination literature suggests a similar pathway conceptualization whereby determinants inform strategy selection, strategies activate mechanisms, and mechanisms produce outcomes [1, 19, 20]. However, while the IRLM is grounded primarily in theories of implementation [21–25], dissemination is fundamentally concerned with communicating information to intended audiences and influencing awareness, understanding, attitudes, and decision-making. As such, dissemination is informed by theories of communication, persuasion, diffusion, consumer psychology, and marketing [2, 26–31], which emphasize how information is designed, communicated, distributed, and received by intended audiences. In line with this theoretical distinction, the literature highlights several components of the dissemination process not explicitly represented in implementation-focused models, including what is being disseminated, the audience, and the channels through which information is communicated [2, 20, 32–37]. Applying the IRLM to a dissemination project therefore risks incomplete specification of these distinct components. It also risks conflating dissemination and implementation processes or outcomes, which is already a challenge in the field [1, 38]. This is evident, for instance, in the case of dissemination outcomes, which are often upstream from implementation outcomes [19, 20] but are not consistently conceptualized or measured as distinct outcomes. Indeed, dissemination and implementation tend to be grouped together under “D&I,” contributing to an under-specification of dissemination constructs that may hinder the advancement of dissemination science as a distinct field. Although numerous valuable resources, frameworks, and toolkits exist to guide dissemination planning and practice [11, 39–42], these resources are primarily designed to help users identify audiences, develop messages, select channels, and plan and evaluate dissemination activities. They do not provide a comprehensive logic model that explicitly specifies the relationships among dissemination components or the causal pathways through which dissemination activities are expected to lead to desired dissemination outcomes.

Informed by work conducted by the Adolescent Medicine Trials Network for HIV Interventions (ATN), we propose the Dissemination Research Logic Model (DRLM) to fill these gaps. Established in 2001 and funded by the National Institutes of Health (NIH), the ATN is the only U.S. national research network focused on adolescents and young adults living with or vulnerable to HIV. The ATN conducts multisite behavioral and biomedical studies across the HIV prevention and care continua, engaging youth most affected by HIV but historically underrepresented in research [43]. In this paper, we describe the components of the DRLM and their sequence, offer six dissemination principles, and apply the DRLM to two ATN projects. We conclude with the model’s anticipated contributions and next steps for refinement. Our goal is to provide a practical tool that visually depicts relationships among dissemination components to strengthen study design, reporting, and synthesis and ultimately advance dissemination research and practice. By providing a dissemination-focused counterpart to the IRLM, the DRLM can enhance methodological precision, comparability, and integration within the dissemination science field.

### DRLM development process

The ATN’s Communications-Dissemination (C-D) Hub began during the prior funding cycle, when the network developed dissemination products across protocols and built an early dissemination infrastructure. Within the current ATN funding cycle, launched in 2023, dissemination became an explicit priority from the outset, and the C-D hub took on the goal of supporting ongoing ATN protocols in planning and evaluating dissemination activities. As this work expanded, we identified a need for a framework that could guide dissemination planning, evaluation, and reporting across diverse ATN studies. We began by conducting a deep dive into the dissemination literature. We extensively reviewed dissemination theories, frameworks, and empirical studies, observing a common emphasis on determinants, strategies, mechanisms, and outcomes as key components of the dissemination process [1, 19, 20, 36, 44, 45]. We therefore used Smith et al.’s IRLM [16] as a starting point for model development. Members of the ATN C-D Hub met regularly over a one-year period to review relevant literature, discuss dissemination concepts, and refine the model. We iteratively adapted the model to incorporate dissemination-specific constructs identified in the literature, including what is being disseminated, the intended audience(s), and dissemination channels. This process resulted in the DRLM. To test and refine its structure, we applied the DRLM to multiple examples within the ATN. Draft versions of the DRLM were repeatedly applied to ATN dissemination activities and revised to improve conceptual clarity, completeness, and usability. Here, we report on the form of the DRLM that resulted from this process.

## Methods

### DRLM structure

**Fig. 1:**
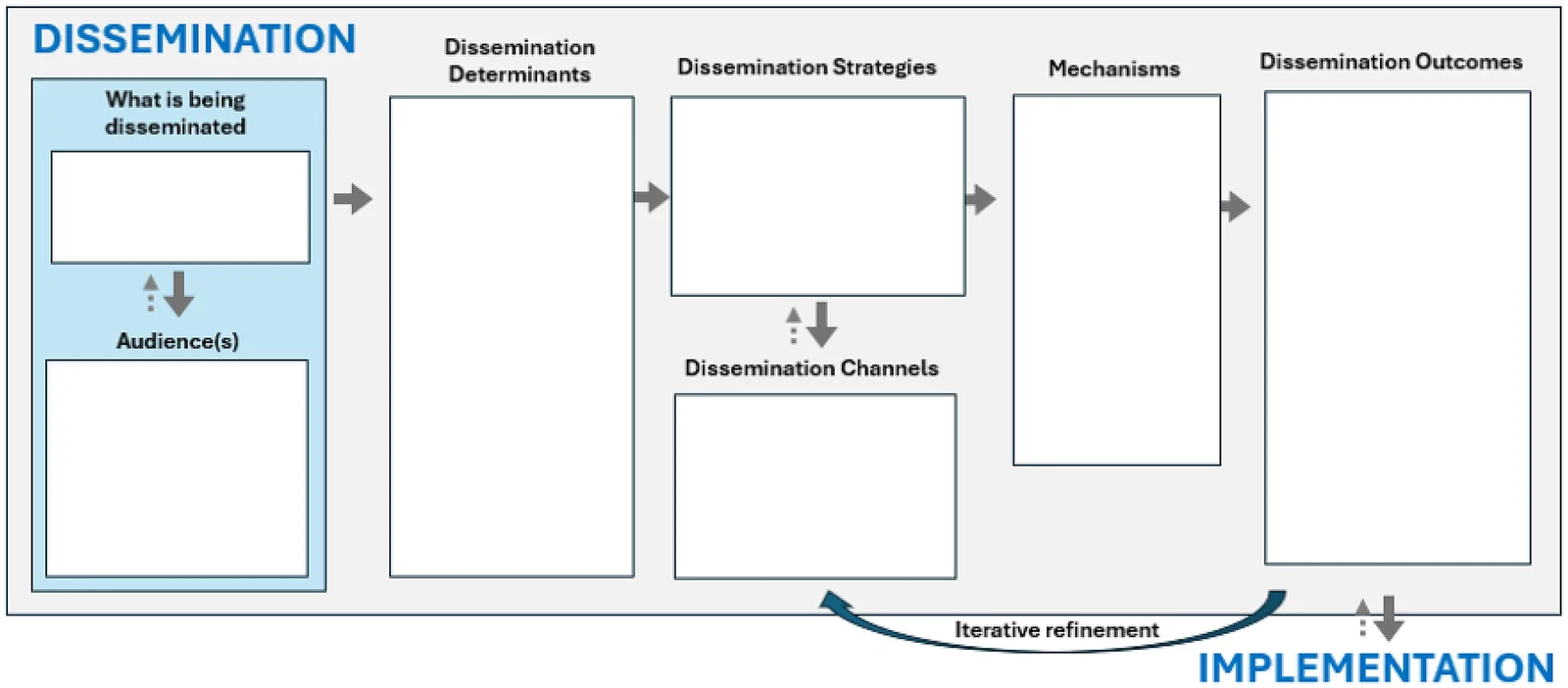
The Dissemination Research Logic Model

Key components of the DRLM include: (1) identifying what is being disseminated; (2) defining the intended audience(s); (3) specifying the contextual determinants that may facilitate or impede dissemination; (4) selecting dissemination strategies that address those determinants and (5) channels through which to disseminate; and (6) articulating the mechanisms and (7) expected dissemination outcomes (Fig. 1).

### DRLM constructs

We proceed to unpack the components of the DRLM, acknowledging that they lack consistent definitions in the literature [1].

*What is being disseminated (“the thing”)*: Consistent with the broad scope of dissemination science, this construct captures the idea, innovation, program, intervention, research finding, treatment, device, service model, policy, guideline, implementation strategy, message, or scientific discovery being disseminated [11, 35, 38]. As Baumann et al. note, most definitions for dissemination describe “the movement or spread of something” [1], highlighting the diversity of content that may be disseminated. As in Baker et al.’s work [19], we can draw on Curran’s simplified terminology for understanding implementation science [46] to refer to this as “the thing” being disseminated.

*Audience*: The audience is the user or intended user of the information or product being disseminated [35, 38]. While dissemination research traditionally emphasizes identifying specific audiences, work in knowledge mobilization and research use shows that evidence moves through networks, relationships, and organizational systems rather than discrete audience groups [1, 35, 38, 47–49]. The DRLM therefore defines audiences broadly as the individuals, groups, or system actors positioned to engage with what is being disseminated, including policymakers, practitioners, patients, community partners, or other end-users [12]. Selecting patients or end-users as the intended audience(s), even when what is being disseminated will primarily be used or implemented by professionals, can be strategic in generating demand for evidence (“pull”) or support downstream implementation efforts [30]. The DRLM can incorporate single or multiple audiences and can be used within broader organizational or system-level efforts that support evidence use.

*Dissemination determinants*: Identifying dissemination determinants underscores the critical importance of understanding the dissemination context [11, 36]. Determinants are features of the context that may influence dissemination processes and outcomes. *Barriers* inhibit or obstruct dissemination efforts, while *facilitators* support or enable these efforts. Determinants may operate at multiple levels, including the characteristics of the information being disseminated, the intended audience, organizations, communities, and broader policy environments. Some dissemination frameworks, such as the Knowledge-To-Action (KTA) Framework [50] and the Bauman framework for international physical activity dissemination [44], explicitly incorporate assessments of dissemination determinants. A community-engaged lens extends this focus on context by emphasizing relational and historical factors that may shape dissemination success, such as prior collaboration, trust, or community readiness [51].

*Dissemination strategies*: We identified a challenge within the dissemination science field around how to depict dissemination strategies, which are often not explicitly defined [35, 52–54] or have multiple definitions (Appendix 1). Dissemination strategies focus on “the how” of dissemination, meaning what we do to support the dissemination process. Common strategies include: using *audience segmentation* (i.e., identifying sub-groups within a population who have similar knowledge, attitudes, or other characteristics relevant to the dissemination effort [2, 12, 55]); *tailoring messaging* (i.e., customizing messages in a way the intended audience can understand and use them [12, 56]); *framing* (i.e., selectively emphasizing certain aspects of the thing to achieve dissemination outcomes for the audience [12, 19]); and *working through influential sources* [12, 47]. Researchers recommend using a package of dissemination strategies [19, 20], which are typically more effective than using single strategies [20]. The growing literature on digital health dissemination highlights the need for additional strategies beyond in-person efforts (e.g., guidance on downloading apps) [57], particularly as technologies, platforms, and user behaviors continue to evolve.

Participatory dissemination strategies directly engage communities in the development and delivery of dissemination activities [58]. Community-engaged dissemination strategies can take a variety of forms, such as co-creating materials or co-planning dissemination meetings, holding reciprocal learning sessions, or enlisting peer or community leaders as messengers to enhance credibility and reach [59]. These strategies align with Purtle et al.’s emphasis on formative audience research [2] and reflect community-based participatory research (CBPR) principles of partnership, equity, and transparency [58].

*Dissemination channels*: In line with Rabin’s definition of dissemination [36], we distinguish dissemination strategies from dissemination channels (sometimes referred to as communication channels [60]), which we define as the communication pathways or routes through which what is being disseminated is delivered to the intended audience(s). Dissemination strategies need channels to work. We group dissemination channels into several broad categories: interpersonal channels (e.g., consultations, meetings, workshops, community forums), professional and scientific channels (e.g., peer-reviewed publications, conference presentations, professional association meetings, webinars, seminars), digital channels (e.g., websites, newsletters, email campaigns, social media platforms), mass media channels (e.g., newspapers, radio, television), and community-based channels (e.g., grassroots events, community partner networks, and text-messaging campaigns) [34, 36, 47, 57, 58, 61]. Different channels vary in their visibility, credibility, interactivity, and suitability for particular audiences and desired dissemination outcomes.

*Mechanisms*: As in implementation science [22], we can think of mechanisms as the processes or events through which a dissemination strategy operates to affect the desired dissemination outcomes [19]. Baker et al. discuss mechanisms as cognitive, emotional, social, or a combination [19]. In a simplified example from their work in school mental health, the dissemination strategy might involve a student sharing a personal story about bullying in the policymaker’s district. This triggers an emotional mechanism—i.e., the policymaker feels motivated to act—and a cognitive mechanism—i.e., the policymaker attends to and gains new knowledge. Together, these mechanisms contribute to the intended dissemination outcome: motivating the policymaker to act in sponsoring or supporting legislation promoting school safety [19].

Mechanisms can be grounded in established theories or frameworks. For example, psychological theories of persuasion and message processing provide useful lenses for understanding how dissemination strategies are intentionally crafted to elicit cognitive and emotional mechanisms [2, 62]. Cognitive and emotional mechanisms often operate together, as in hot cognition, where affect influences how audiences (particularly adolescents) attend to and act on evidence, which we treat as a mechanism rather than a dissemination outcome [63, 64]. These perspectives reinforce that the same strategy can operate through multiple pathways, shaping how audiences attend to, interpret, and act on information [2].

*Dissemination outcomes*: Dissemination outcomes are the effects of what is being disseminated on the intended audience(s) [65]. There are different views in the literature on what constitutes a dissemination outcome. Baker et al. describe dissemination outcomes as changes to antecedents of behavior change (e.g., awareness, knowledge, perceptions, motivation) [19]. Purtle et al. propose the possibility for proximal and distal dissemination outcomes, where proximal outcomes include the antecedents of behavior change and distal outcomes can include behaviors [12]. Baumann et al. identify other dissemination outcomes defined by researchers, including knowledge utilization, changes in policy, adoption, fidelity, maintenance, and cost [1]. Readers seeking conceptual lists of dissemination outcomes can consult Baumann et al.‘s catalog of constructs compiled across dissemination frameworks [1], Leeman et al.‘s focus on awareness, knowledge, attitudes, and intention to adopt [25], and our taxonomy of dissemination outcomes developed in the context of HIV prevention research [66]. These perspectives differ in where dissemination ends and implementation begins. Dissemination strategies work primarily on motivation: awareness, knowledge, perceptions, and intentions. We locate the boundary at the decision to implement, a formal commitment to act on the evidence. Outcomes up to and including that commitment are dissemination outcomes; the behaviors that follow, such as adoption, are implementation outcomes [23]. From this view, the distal behavioral outcomes Purtle et al. describe are better understood as implementation outcomes [9], consistent with Baker et al.‘s motivational-versus-behavioral distinction. Our dissemination outcomes taxonomy also offers sample measures for each dissemination outcome, such as download counts for exposure or intention scales for action readiness [66].

### Activity sequence, adaptation, and resources

The DRLM is presented with arrows denoting a sequence for clarity (Fig. 1). Identifying what is being disseminated and the audience(s) in the blue box on the left is a foundational step that informs the rest of the dissemination process. The arrow sequence between the two boxes reflects that the dissemination process often begins by defining what is being disseminated, which informs the intended audience(s), but it can be easiest to begin with the intended audience and then define what to disseminate [35, 47]. In some knowledge mobilization or organizational research-use approaches, the starting point may reflect catalysts, capacities, or system-level priorities rather than a discrete audiences. For example, the SPIRIT Action Framework [49] and its recent adaptation for academic-policy engagement [48] organize research use into domains of catalysts, organizational capacities, engagement actions, and research-use outcomes, providing conceptual lists that readers can draw on when their dissemination effort begins from system-level priorities rather than a defined audience. These foundational decisions shape the identification of dissemination determinants, which inform the selection of dissemination strategies and associated channels to address the barriers and leverage facilitators. The strategies operate through mechanisms, which lead to dissemination outcomes.

Dissemination outcomes are thought to precede the implementation process given that effective dissemination can promote readiness for implementation [12, 19, 32], which can lead to downstream changes in individual health outcomes [16, 23]. In practice, dissemination is rarely a linear process [1, 34, 35, 50, 67]. Dissemination and implementation often unfold simultaneously, with outcomes feeding back to shape determinants, strategies, and channels in iterative cycles. Dissemination and implementation can also occur simultaneously and support each other in the uptake of evidence-based interventions [1, 67]. This dynamic process can resemble chaos theory, where small shifts in context or credibility may cascade into large effects on reach, uptake, or sustainability [68, 69].

As with the IRLM, the DRLM is meant to be flexible such that users can adapt their starting point and the ordering of the constructs as needed or focus on only those outcomes relevant to a project. The DRLM invites users to view dissemination as a cyclical process of learning and relationship-building in which communities and researchers co-create, share, and reinterpret evidence over time. For example, users may choose to organize the dissemination determinants box by subdomains (e.g., innovation-, organizational-, community-, or system-level factors), similar to adaptations proposed for the IRLM [16]. Likewise, dissemination efforts often involve audiences operating across multiple levels (e.g., policymakers, organizations, providers, and community members), and users may choose to organize DRLM components to reflect these multilevel dissemination pathways, consistent with growing attention to multilevel processes in implementation science [70]. Users may also choose to explicitly map relationships among specific dissemination determinants, strategies, channels, mechanisms, and outcomes. Building on approaches proposed for the IRLM [16] and Baker et al.’s empirical example using causal logic [19], such specification may strengthen transparency and make hypothesized dissemination pathways more explicit. Because dissemination efforts frequently involve multiple audiences, overlapping pathways, and interactions across settings, however, the most useful approaches for representing these relationships remain an area for future methodological development.

Appendix 2 provides a fillable PDF version of the DRLM. A teaching version is in Appendix 3. Appendix 4 presents a dissemination planning worksheet template that specifies audience-specific dissemination determinants, strategies, channels, mechanisms, and outcomes. The worksheet demonstrates how a single dissemination effort may require distinct dissemination pathways for different audiences and provides one example of how the DRLM can be translated into a pragmatic dissemination planning tool using a matrix format. Users of the DRLM may identify other useful practical tools for its operationalization.

### Dissemination principles

The literature highlights numerous dissemination principles and best practices [11, 34, 71–73], which are well-suited to underpin the creation of one’s DRLM. These principles emerged from our review of dissemination theories, frameworks, empirical studies, and dissemination guidance documents. Unlike the IRLM whose principles focus primarily on use of the model itself [16], the following principles synthesize best practices for conducting dissemination. As with the IRLM [16], we offer these as flexible principles rather than prescriptive rules. They are intended to inform how users develop, plan, and evaluate dissemination activities using the DRLM across diverse projects and contexts. We organize these into six principles.

*Principle 1: Plan and resource dissemination from the start.* It is important to invest in dissemination from the start of a project and to garner institutional support, including from funders, to ensure efforts are adequately resourced [36, 74]. Funders must acknowledge the costs of dissemination and encourage the inclusion of dissemination efforts in budgets, as reflected in NIH clinical trial requirements [34]. When considering the costs and benefits of dissemination, it is worth deciding whether to focus dissemination efforts on a single study or multiple studies. Wilson argues that not every study needs to be widely disseminated [75].

*Principle 2: Decide who is responsible for dissemination.* Deciding who the disseminator will be is essential. While some argue that the disseminator should be individuals other than researchers [72], we adopt the viewpoint that in a research project, the responsibility for dissemination is at minimum with the research team but that all those invested in the dissemination effort should be included in the dissemination process [11, 34], similar to what has been argued for implementation science [76]. We recognize the misalignment of incentives for researchers, which generally reward academic dissemination (e.g., peer-reviewed publications, conference presentations) above non-academic forms of dissemination [61]. Incorporating CBPR and community-engaged approaches helps address this misalignment by reframing dissemination as a shared responsibility between researchers and community partners—one that values co-ownership of findings, mutual learning, and reciprocal exchange [59, 77].

*Principle 3: Consider the source.* In the process of determining what to disseminate, we recommend considering what Brownson et al. call the *source*—i.e., the individual, group, or organization originating the message being disseminated [35, 38]. Sources may include researchers or academic groups, government bodies, or expert groups of researchers and practitioners [38]. The credibility, trustworthiness, expertise, and identity of the messenger, as well as the degree of alignment between the source and audience values, can influence how information is received, interpreted, and acted upon [27, 78–80].

*Principle 4: Choose a guiding framework.* Deciding on a dissemination framework is critical [11, 72], as in implementation science [81]. A guiding framework supplies the determinants, mechanisms, and outcomes that users populate within the DRLM, anchoring the model in established theory. While frameworks for dissemination are less numerous than for implementation, several do exist but remain underutilized. In Baumann et al.’s work, only 51% of empirical papers reviewed used a framework to guide their dissemination study [1]. Users of the DRLM are encouraged to consult reviews highlighting available frameworks from which to choose [1, 77, 82, 83]. The choice of framework should be guided by the dissemination context, audience(s), and desired dissemination outcomes, as different frameworks emphasize different determinants, mechanisms, and outcomes. Because dissemination science is still maturing and dissemination-specific frameworks remain relatively underdeveloped, it may be useful to adapt implementation-focused frameworks for dissemination projects as some scholars have begun to do (e.g., RE-AIM- [84], EPIS- [2], CFIR- [85]).

*Principle 5: Ground dissemination in formative research and evaluation.* Conducting formative, data-driven planning, as emphasized by Purtle et al. [2], is also essential to dissemination research. Formative audience research, which seeks to characterize an audience’s awareness, adoption, attitudes, and preferences [2], and audience segmentation research, which aims to understand how to tailor materials to different audience groups [2], can inform dissemination strategies, channels, and mechanisms. Undertaking these formative research activities through participatory processes across the lifespan of a DRLM helps to ensure that dissemination efforts remain responsive and equitable. Systematically evaluating dissemination efforts provides critical feedback to refine strategies and enhance impact over time [34, 71].

*Principle 6: Engage community members and partners throughout.* Engaging community members and partners throughout the process—an element too often overlooked [72, 77] but which is gaining more attention [61]—also helps ensure that dissemination is relevant and that what is being disseminated can be accessed equitably [11, 34, 36, 54]. Researchers are encouraged to involve multiple types of individuals in the design and dissemination process, including study participants, through such avenues as participatory and co-design methods [11, 34]. Establishing continuous, bi-directional communication (e.g., through a steering committee) further strengthens collaboration and alignment [71]. Building community trust through transparent and ethical practices is essential, as is tailoring dissemination products to multiple audiences and employing diverse media to expand reach [71]. The Designing for Dissemination (D4D) approach, for instance, highlights the importance of equity, fit, and early partnership and engagement while emphasizing planning for dissemination from the very beginning of a project [11]. Thomas et al. illustrate how audiences can be involved during the active dissemination process itself, for example, by being asked to real aloud short extracts from interviews or generating and sharing aloud images to facilitate discussion on findings at a dissemination event [86].

### Overview of two ATN studies

Our research process involved applying the DRLM to two ATN studies. Project PrEPare (ATN 113) was the first U.S. trial of PrEP among sexual minority males aged 15–17 years vulnerable to HIV. Across six sites, 78 participants received daily tenofovir disoproxil fumarate/emtricitabine (TDF/FTC), behavioral counseling, and routine HIV and sexually transmitted infection (STI) prevention services. PrEP was safe and well-tolerated, with no serious adverse events. Three HIV infections occurred with sub-protective drug levels, and sexual risk behaviors did not increase despite high background STI prevalence [87]. ATN 113 demonstrated the feasibility and safety of PrEP for adolescents, and the recruitment and retention potential for adolescents in biomedical HIV prevention research, while underscoring the need for adolescent adherence support [87].

Tailored Motivational Interviewing (TMI; ATN 128 and ATN 146) is a provider-level adjunct intervention that adapts motivational interviewing (MI)—a collaborative conversation style for strengthening individual motivation and commitment to change [88]—for HIV-related behaviors and clinical context to improve staff communication skills in adolescent HIV clinics [89]. In ATN 128, community health workers receiving a two-day workshop plus structured coaching significantly improved their MI competence, with gains sustained for two years [90]. ATN 146 extended TMI across 10 clinics with 151 providers, demonstrating substantial, durable improvements in MI competence and fidelity [89]. Together, these studies show that modest training and coaching can meaningfully enhance provider communication skills in real-world adolescent HIV care.

## Application of the DRLM to two ATN studies

### Project PrEPare (ATN 113)

**Fig. 2:**
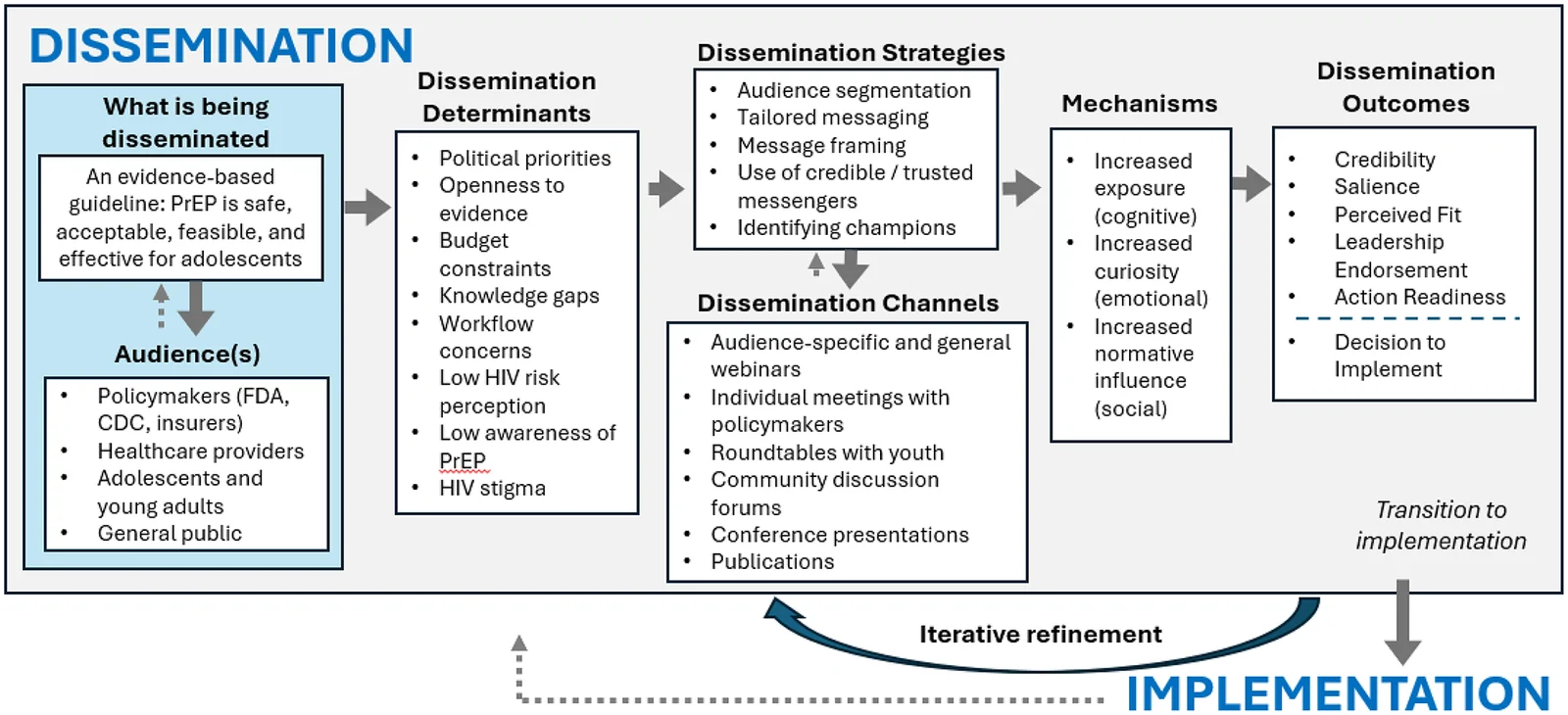
The Dissemination Research Logic Model applied to Project PrEPare (ATN 113)

The Project PrEPare example (Fig. 2) began with *what was being disseminated*: a guideline based on ATN 113 findings that PrEP is safe, acceptable, and feasible, and effective in preventing HIV for adolescents with high medication adherence [87]. The specified *audiences* were policymakers, healthcare providers, and youth. *Dissemination determinants* for policymakers included competing political priorities, openness to evidence, and budget constraints. Determinants for providers included knowledge gaps in prescribing PrEP to adolescents and workflow concerns. Determinants for youth included low awareness of PrEP and low HIV risk perception. HIV stigma was a determinant for all groups. Applying the DRLM retrospectively, we identified a package of *dissemination strategies* that likely supported dissemination of the findings. This included audience segmentation (i.e., specifying each audience of interest), tailoring messaging to each audience, message framing specific to each audience, the use of credible or trusted messengers, and the identification of champions. The Project PrEPare team used several *dissemination channels*: expert consultations, webinars and seminars, roundtables with youth, conference presentations, peer-reviewed publications, and community discussion forums. For policymakers, presentations, seminars, and expert consultations increased exposure to evidence supporting adolescent PrEP and facilitated discussion of regulatory and financing implications. For healthcare providers, professional presentations and publications increased awareness of the safety, feasibility, and effectiveness of PrEP for adolescents and its relevance to clinical practice. For youth, roundtables and community discussion forums provided opportunities to learn about PrEP, discuss questions and concerns, and consider its relevance as an HIV prevention strategy. Across audiences, these dissemination activities likely operated through *cognitive mechanisms* (e.g., attention and processing the evidence), *emotional mechanisms* (e.g., curiosity and interest), and *social mechanisms* (e.g., normative social influence), which in turn contributed to *dissemination outcomes*. Following the dissemination outcome sequence [66], these audiences moved from encountering and understanding the evidence to judging its fit, endorsing it, and ultimately deciding to implement it.

The decision to implement is the terminal dissemination outcome that likely contributed to several downstream policy changes, including: (1) a change in policy through the U.S. Food and Drug Administration (FDA)’s expanded licensure of tenofovir disoproxil fumarate/emtricitabine (TDF/FTC) for adolescent PrEP; (2) updates to Centers for Disease Control and Prevention (CDC) guidelines to recommend PrEP for adolescents; and (3) updates to insurance coverage policies to cover PrEP for adolescents. These policy changes helped create conditions that could facilitate greater adolescent access to PrEP and support broader implementation of PrEP services. This example illustrates how the DRLM can be used to conceptualize dissemination processes through which evidence from a single study may influence policy, practice, and ultimately population health.

### Tailored motivational interviewing (ATN 128 and ATN 146)

The TMI example illustrates dissemination of an intervention to a single audience, using the Exploration-Preparation-Implementation-Sustainment (EPIS) framework to identify determinants across multiple domains. It also shows how dissemination and implementation can interact over the life an intervention. TMI was developed, implemented, and studied within the ATN [91, 92]. The team is now disseminating TMI more broadly to reach clinic leaders who are not yet aware of it. Where dissemination succeeds, implementation is expected to prompt new clinics to implement TMI. Hence, this example centers on dissemination while showing how it follows from and leads back to implementation, thus depicting the dynamic interplay between dissemination and implementation.

**Fig. 3:**
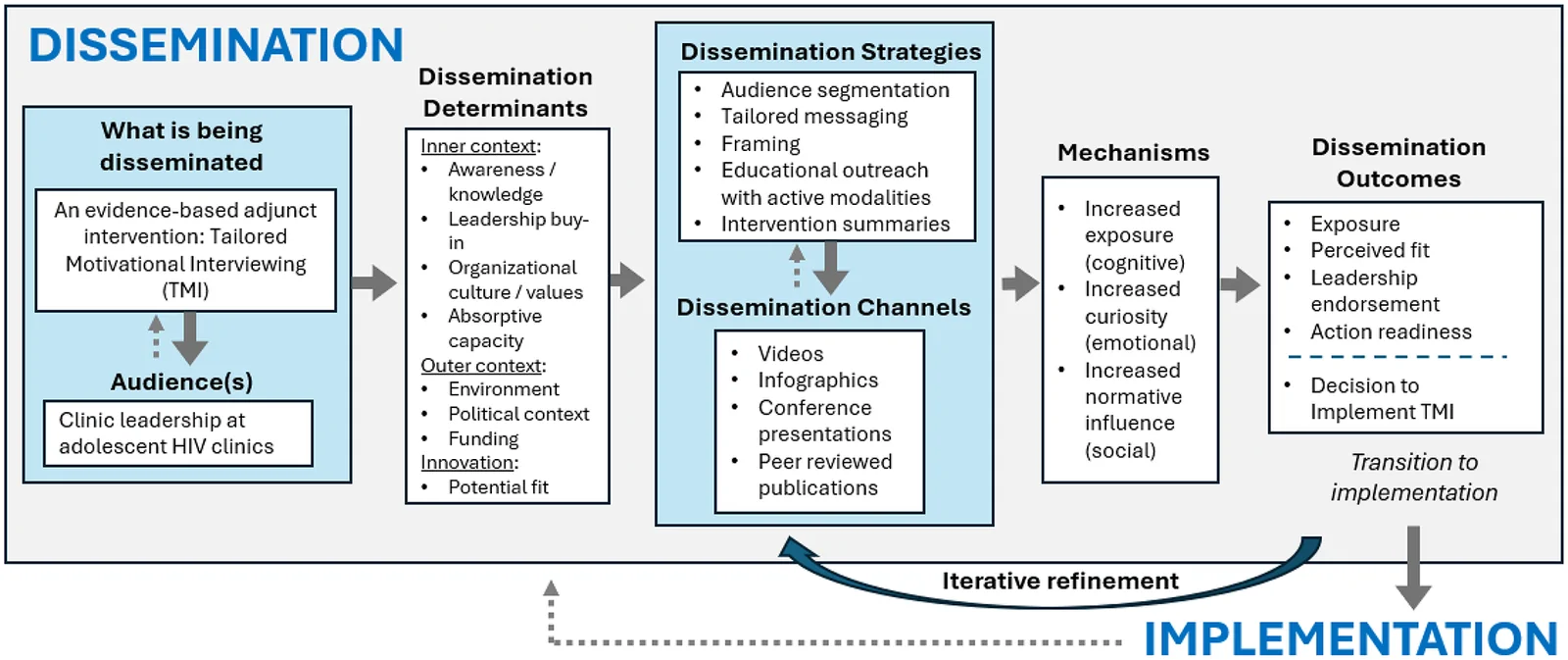
The Dissemination Research Logic Model applied to tailored motivational interviewing (TMI; ATN 128 and 146)

As shown in Fig. 3, the TMI intervention is *what is being disseminated* and clinic leadership in adolescent HIV clinics embody *the audience*. Because the team used the EPIS framework [91, 92], an implementation framework, determinants are organized according to the EPIS domains. Within the inner context, *dissemination determinants* include awareness of and motivation to use TMI, leadership buy-in, organizational culture and values, and absorptive capacity—i.e., the ability to assimilate TMI into clinics. Within the outer context, determinants include the environment (i.e., the allocation of resources that affect clinic practice/intervention implementation), the political context (i.e., the extent of political support for initiatives like TMI), and funding (i.e., the degree of funding available for TMI and similar interventions). Innovation-related determinants include the degree of fit of TMI within the clinic system. To address these determinants, the ATN team has developed package of *dissemination strategies*, including: audience segmentation (i.e., focusing on clinic leadership); tailored messaging to emphasize TMI’s efficiency, scalability, and alignment with clinics’ goals of supporting youth autonomy; framing TMI as a translational bridge between behavioral science and clinical communication; educational outreach with active modalities, such as monthly free one-hour “tasters” of TMI; and intervention summaries. *Dissemination channels* used to date include videos, infographics, websites (e.g [93, 94]), compendiums (e.g., via the Health Resources and Services Administration-HRSA, CDC), email and listservs, a cross-disciplinary and community-based mailing list, conference presentations, and peer-reviewed publications. Dissemination strategies are hypothesized to operate through the *cognitive mechanism* of increased exposure to TMI, the *emotional mechanism* of increased curiosity about TMI, and the *social mechanism* of increased normative influence (i.e., greater receptivity by seeing other leaders engage with TMI). Desired *dissemination outcomes* include increased exposure, perceived fit, leadership endorsement, action readiness, and ultimately decision to implement TMI.

## Discussion

The DRLM responds to calls to strengthen the conceptual clarity and transparency of dissemination science research [1–3, 34]. While the broader D&I field has expanded rapidly, the dissemination science field lacks a standardized, field-tested tool to specify and link its core components, as the IRLM offers the implementation science field [16]. The DRLM fills this gap by providing a structured but flexible framework that clarifies causal pathways, integrates multiple components into a single visual, and supports both conceptual precision and practical usability. In addition to supporting dissemination planning and reporting, the DRLM can be used to prospectively specify measurable dissemination outcomes and corresponding evaluation metrics, facilitating more rigorous assessment of dissemination effectiveness across studies. It is intended to work alongside rather than replace broader approaches that focus on knowledge mobilization, knowledge translation, and research use [48, 49], offering a more granular specification of dissemination activities that can be nested within relational and system-level efforts. It is also intended to complement existing dissemination planning resources [11, 39–42] by visually specifying how what is being disseminated, audiences, dissemination determinants, strategies, channels, mechanisms, and outcomes are hypothesized to relate to one another. In this paper, we introduced the DRLM and applied it to two ATN studies, demonstrating its utility and suggesting future adaptations. Like the IRLM [16], the DRLM supports rigorous study design, reporting, and synthesis in dissemination science.

The DRLM has several notable strengths. First, it provides visual clarity by helping researchers, practitioners, and community partners collectively specify how change is expected to occur and why specific dissemination activities are being pursued [16, 95–97]. This approach complements calls for planning for dissemination from project inception [11] and facilitates co-learning among diverse partners consistent with CBPR principles. Second, the DRLM integrates key dissemination components identified in the literature [1, 12, 19] into one framework. Third, the DRLM is flexible, supporting use across policy, clinic, and community settings and multiple dissemination pathways. We encourage users to adapt the boxes, sequence, and terminology to fit their context. Fourth, the DRLM is meant to facilitate collaboration across diverse partners in planning and carrying out dissemination projects. We hope its visual structure, alongside the practical tools provided in the appendices, will support these collaborative efforts using CBPR principles. Together, these characteristics make the DRLM a useful tool for strengthening planning, measurement, and evaluation—areas that remain underdeveloped in dissemination science [1, 12].

Our application of the DRLM to two ATN studies revealed conceptual challenges that may arise for users, particularly in distinguishing among the components of the dissemination process. Dissemination strategies and dissemination channels are frequently used interchangeably in the literature despite representing distinct components of the dissemination process. Further work is needed to clarify their conceptual boundaries and to develop comprehensive classifications of dissemination strategies and channels that can be applied consistently across studies. Mechanisms and outcomes are also easily conflated, since the cognitive processing that a strategy sets in motion is a mechanism whereas the understanding it produces is an outcome. Differentiating dissemination and implementation outcomes may be particularly complex. In the TMI example, the dissemination outcome is to the decision to implement TMI; adoption, which follows that decision, is an implementation outcome [23]. Locating the boundary at this decision to implement clarifies this distinction. More work is needed to clarify these components, building on our team’s dissemination outcomes taxonomy [66]. Additionally, designing studies to measure the outcomes in the DRLM may pose challenges. Outcomes may occur over an extended period, necessitating multiple studies. Future work should develop and test hybrid studies that intentionally integrate dissemination outcomes with effectiveness and implementation outcomes [98].

Our work on the DRLM is at an early stage, and there are multiple opportunities to shape and refine the model going forward. We are currently using the DRLM to inform dissemination activities for six ongoing ATN studies, which will facilitate further learning. Next, we plan to engage in a range of dissemination audiences, such as policymakers, practitioners, and community partners, to assess the model’s structure and utility and to gather feedback on usability, clarity, and fit across settings. We also see value in comparing DRLM-based dissemination planning with IRLM-based approaches to clarify where each model is most useful, and in developing measures for the dissemination outcomes the model specifies. We also plan to focus on dissemination of the DRLM itself by identifying key audiences, developing practical tools and training materials, soliciting user feedback, and assessing decisions to use the model across diverse contexts.

Several limitations warrant consideration. The Project PrEPare example is retrospective and thus was not informed by a dissemination framework. The DRLM has thus far been developed and applied only within the ATN context and to two illustrative cases: Project PrEPare, a multi-audience dissemination effort related to adolescent PrEP, and TMI, a clinic-based dissemination effort pursuing adoption of an evidence-based intervention. While the DRLM offers a structured approach, it does not prescribe specific theories, frameworks, or measures, which may lead to variability in how teams operationalize and evaluate its components. Broader testing is needed to assess applicability across topics, designs, and resource levels. Because logic models can oversimplify the dynamic, non-linear nature of dissemination [96], the examples presented in this paper are intended to illustrate application of the DRLM rather than provide comprehensive reconstructions of dissemination processes, which often occur simultaneously across multiple audiences, channels, and levels. We encourage users to document feedback loops and adaptations over time to preserve transparency and validity. Finally, future work should empirically test the DRLM in varying contexts, engage multiple audiences to assess usability, and evaluate whether its use strengthens study rigor, comparability, and equity in dissemination outcomes.

## Conclusion

As with the IRLM, the DRLM is not static or prescriptive template. Rather, it is a living, iterative tool designed to capture and make sense of the dynamic, nonlinear processes that characterize dissemination. The relationships among what is being disseminated, the intended audience(s), determinants, strategies, channels, mechanisms, and outcomes evolve over time and across settings, reflecting the realities of dissemination as both a science and a practice. To sustain this kind of iterative learning, research networks that use logic models to coordinate activities across projects, such as multisite collaboratives and research centers, offer a useful model [99]. Embedding the DRLM within such structures could facilitate shared learning about dissemination processes and provide insight into how the DRLM itself evolves through use. As researchers and practitioners apply the DRLM across projects, ongoing feedback and refinement will clarify its generalizability and strengthen its utility as a planning and evaluation tool. By offering a shared visual language for articulating these relationships, the DRLM has the potential to enhance conceptual clarity, promote equitable and participatory dissemination practices, and accelerate the translation of research into real-world impact. Ensuring that the DRLM continues to evolve through use, collaboration, and critique will help it remain a relevant and inclusive resource for the dissemination science community.

## Supplementary Information

Below is the link to the electronic supplementary material.

Supplementary Material 1

Supplementary Material 2

Supplementary Material 3

Supplementary Material 4

## Data Availability

Not applicable.
