# Supplementary material for: The Dissemination Research Logic Model for advancing dissemination science and practice
Source: Implement Sci. 2026 Jul 30;21:61. doi: 10.1186/s13012-026-01530-2 (PMC13711734; doi:10.1186/s13012-026-01530-2)
Supplement: Supplementary file 1 [file 13012_2026_1530_MOESM2_ESM.pdf]

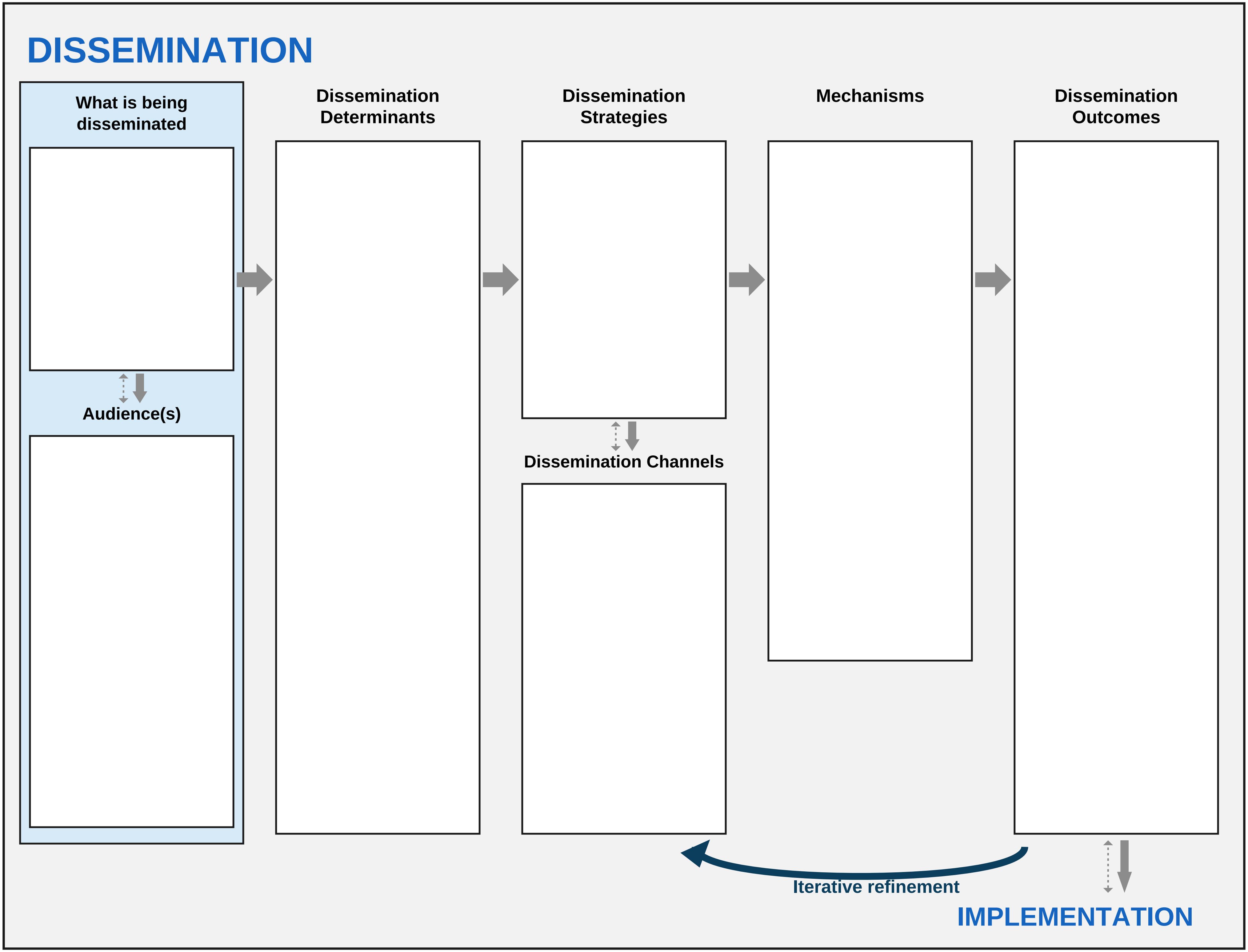
DISSEMINATION
What is being disseminated
Audience(s)
Dissemination Determinants
Dissemination Strategies
Dissemination Channels
Mechanisms
Dissemination Outcomes
Iterative refinement
IMPLEMENTATION
