# Supplementary material for: The Dissemination Research Logic Model for advancing dissemination science and practice
Source: Implement Sci. 2026 Jul 30;21:61. doi: 10.1186/s13012-026-01530-2 (PMC13711734; doi:10.1186/s13012-026-01530-2)
Supplement: Supplementary file 2 [file 13012_2026_1530_MOESM1_ESM.docx]

**Supplementary Table 1**: A selection of sources, definitions, and examples of dissemination strategies

| Source | Definition | Examples |
| --- | --- | --- |
| Baker et al. [19] | Intentional methods used to communicate strategically crafted information about an evidence-based practice to specific audiences to alter antecedents of behavior change (e.g., awareness, knowledge) in ways that increase the likelihood of role-specific implementation behaviors. | - Using audience segmentation - Drawing on multiple media formats - Tailoring messaging - Ensuring messages are responsive to local norms (e.g., culture, language) - Using “plain” language - Incorporating visualizations (e.g., infographics); - Personalizing risk information |
| Baumann et al. [1] | The approaches or actions of a dissemination process | - Assessing dissemination determinants - Adapting the dissemination object (i.e., “the thing”) to the context - Modifying organizational supports or resources to enable dissemination - Monitoring and evaluating the dissemination process - Increasing the audience’s skills to facilitate adoption or uptake |
| Leeman et al. [46] | Any action or set of actions that target public health and healthcare decision makers’, clinicians’, and other staffs’ awareness, knowledge, attitudes, and intention to adopt an evidence-based intervention | When developing messages/materials:   - Using audience segmentation, tailoring, and framing   When distributing messages and materials:   - Working through trusted, accessible channels - Enlisting knowledge brokers or “gatekeepers” - Aligning dissemination methods with audience preferences |
| Ashcraft et al. [51] | The process of considering the source, message, audience, and channel of a dissemination effort (this definition is implied) | For engaging policymakers:   - Engaging policymakers early and throughout the research process - Building coalitions and external support - Using evidence “champions” or “brokers” - Ensuring evidence is timely, relevant, and contextually appropriate - Making research products concise accessible - Understanding the policy process and key actors |
| Dadgostar et al. [97] | Actions and processes that promote and support the dissemination of information, conveying it from a source to the audience through the intentional development and selection of sources, content, and channels. | For dissemination via social media:   - Using one-way spread (just posting content on social media platforms) - Invoking conversations (e.g., creating online communities or expert Q&As) - Promoting peer motivation (e.g., facilitating the sharing and feedback among users) - Partnering with influencers or opinion leaders to amplify reach |
