# Supplementary material for: The Dissemination Research Logic Model for advancing dissemination science and practice
Source: Implement Sci. 2026 Jul 30;21:61. doi: 10.1186/s13012-026-01530-2 (PMC13711734; doi:10.1186/s13012-026-01530-2)
Supplement: Supplementary file 3 [file 13012_2026_1530_MOESM4_ESM.docx]

# Appendix 4: Dissemination Research Logic Model (DRLM) Planning Template

Use this worksheet to map one or more dissemination pathways, including audience-specific determinants, dissemination strategies, channels, mechanisms, and dissemination outcomes.

User Instructions:

- Customize the number of rows based on the number of audience groups, dissemination pathways, or dissemination activities being planned.
- The template may be completed at varying levels of detail depending on project needs. Users may choose to develop a single pathway or multiple pathways for different audiences.
- Where feasible, consider explicitly linking determinants, strategies, channels, mechanisms, and outcomes across columns (e.g., using matching numbers, letters, colors, rows, or other notation). Such alignment can help clarify hypothesized causal pathways and make dissemination planning more transparent.
- Users may adapt the structure, terminology, and sequencing of the template to fit their dissemination context.

Specify what is being disseminated (“The thing”): __________________________________________

________________________________________________________________________________________

**DRLM Operational Matrix**

| Audience(s)  *(Which audience(s) need to receive it?)* | Dissemination Determinants  *(What barriers and facilitators influence dissemination?)* | Dissemination Strategies  *(What tools/ approaches can support dissemination?)* | Dissemination Channels  *(Through which vehicles/mediums will dissemination occur?)* | Mechanisms  *(How do strategies influence outcomes?)* | Dissemination Outcomes  *(What changes will result from dissemination?)* |
| --- | --- | --- | --- | --- | --- |
|  |  |  |  |  |  |
|  |  |  |  |  |  |
|  |  |  |  |  |  |
|  |  |  |  |  |  |

## Transition to Implementation

Describe how dissemination outcomes may support or transition into implementation processes:

- Identify the specific implementation processes (e.g., adoption, clinic-wide adaptation, integration) that are unlocked by achieving these dissemination outcomes:
- List the key implementation partners or champions who will take ownership during this transition hand-off:

## Iterative Refinement Notes

Document adaptations, refinements, audience feedback, and changes to dissemination strategies or channels over time:

- Date / Phase of Study:
- Observed Challenge or Audience Feedback:
- Resulting Adaptation made to Strategy/Channel (Include justification):
